# Unconscious processing of invisible visual stimuli

**DOI:** 10.1038/srep38917

**Published:** 2016-12-12

**Authors:** Chen Song, Haishan Yao

**Affiliations:** 1 Institute of Cognitive Neuroscience, University College London, London WC1N 3AR, UK; 2 Wellcome Trust Centre for Neuroimaging, University College London, London WC1N 3BG, UK; 3 Department of Psychiatry, University of Wisconsin - Madison, Madison 53719 WI, USA; 4 Institute of Neuroscience and State Key Laboratory of Neuroscience, Shanghai Institutes for Biological Sciences, Chinese Academy of Sciences, Shanghai 200031, China

## Abstract

Unconscious processing of subliminal visual information, as illustrated by the above-chance accuracy in discriminating invisible visual stimuli, is evident in both blindsight patients and healthy human observers. However, the dependence of such unconscious processing on stimulus properties remains unclear. Here we studied the impact of stimulus luminance and stimulus complexity on the extent of unconscious processing. A testing stimulus presented to one eye was rendered invisible by a masking stimulus presented to the other eye, and healthy human participants made a forced-choice discrimination of the stimulus identity followed by a report of the perceptual awareness. Without awareness of the stimulus existence, participants could nevertheless reach above-chance accuracy in discriminating the stimulus identity. Importantly, the discrimination accuracy for invisible stimuli increased with the stimulus luminance and decreased with the stimulus complexity. These findings suggested that the input signal strength and the input signal complexity can affect the extent of unconscious processing without altering the subjective awareness.

Visual information outside of awareness can affect conscious experience^1^,^2^, motor response^3^,^4^, and even goal pursuit^5^,^6^. To understand the power and limits of subliminal visual information, it is necessary to address the degree to which supraliminal visual information can be processed. If observers can correctly “guess” the identity of visual stimuli despite being unaware of the stimuli, it is intuitive that such subliminal visual information may affect behavior in a fashion similar to the way supraliminal visual information influences behavior^7^. The unconscious processing of subliminal visual information has been reported in blindsight patients, who, due to the lesions in primary visual cortex, cannot consciously perceive visual stimuli in their defect visual field, but can nonetheless correctly discriminate the stimuli^8^. This unconscious processing of subliminal visual information is also evident in healthy human observers, where visual stimuli rendered invisible can be discriminated at above-chance accuracy^9^.

Despite these established dissociations between perceptual awareness and correct discrimination of visual stimuli, it remains unclear how the unconscious processing of invisible visual stimuli is dependent on the stimulus properties. We suggest that the extent of unconscious processing may be affected by the strength and the complexity of the signal carrying subliminal visual information, which may in turn determine the degree towards which subliminal visual information can influence behavior. To test this hypothesis, we investigated the influence of stimulus luminance and stimulus complexity on the extent of unconscious processing. Using the paradigm of continuous flash suppression, we rendered a testing stimulus invisible by presenting it to one eye while presenting a masking stimulus to the other eye^3^,^10^. This created situations where the same testing stimulus was totally invisible in some trials yet fully or partially visible in other trials^2^, possibly due to the fluctuations in cortical signal evoked by the testing stimulus.

Such induction of different perceptual awareness scales by the same physical stimulus allowed us to compare the discrimination accuracy between trials where the testing stimulus was invisible and visible, respectively. We found that in trials where the testing stimulus was invisible, participants could nonetheless reach above-chance discrimination accuracy. Based on this observation, we explored how the discrimination accuracy of invisible testing stimulus changed with the luminance and the complexity of the stimulus. To vary the stimulus complexity, we compared simple, low-level visual stimuli such as oriented grating^11^, with complex, high-level visual stimuli such as face or house^12^. We found that while the testing stimulus remained invisible, the discrimination accuracy increased with the stimulus luminance and decreased with the stimulus complexity. Our findings suggested that the strength and the complexity of the signal carrying subliminal visual information can affect the extent of unconscious processing without altering the subjective awareness.

## Material and Methods

### Participants and Apparatus

Twelve healthy volunteers gave written informed consent to participate in this study that was approved by the Biomedical Research Ethics Committee of the Shanghai Institutes for Biological Sciences. The study and methods were carried out in accordance with the guidelines of the Biomedical Research Ethics Committee of the Shanghai Institutes for Biological Sciences and the guidelines of the declaration of Helsinki. The participants were young adults (aged 19 to 25, 8 females, 4 males) with normal or corrected-to-normal vision and no neurological history. Apart from one of the authors (CS), all participants were naive to the aims of this study and received payment for participation. The experiments were programmed in MATLAB using Psychtoolbox^13^ and conducted in a darkened room with the monitor providing the only significant source of light.

### Stimuli and Procedure

We measured the discrimination accuracy of visual stimulus in conditions where participants were unaware, partially aware, or fully aware of the stimulus. For this purpose, we presented a low-luminance gray-scale testing stimulus to one eye and a high-contrast colorful mask to the other eye, while the eye-of-presentation was random and counter-balanced across trials. To test the influence of stimulus luminance and stimulus complexity, we conducted three separate experiments using simple or complex stimuli at one of five possible luminance levels. The testing stimulus was a gray-scale sinusoidal grating (spatial frequency: 2.8 cycles per degree of visual angle) oriented at 45 degree towards left or right in experiment one, a gray-scale female face or house in experiment two, and a gray-scale happy or sad cartoon face in experiment three. The masking stimulus was a high-contrast colorful Mondrian pattern flashed at a frequency of 33.33 Hz in all three experiments.

**Figure 1:**
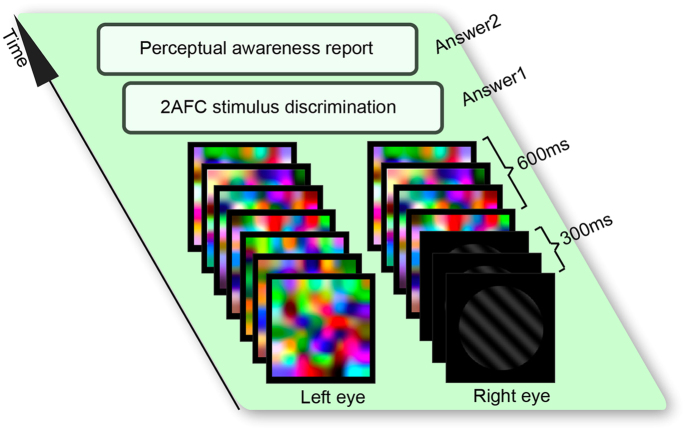
Schematic depiction of experimental paradigm. A low-luminance gray-scale testing stimulus presented to one eye was rendered invisible by a high-contrast colorful masking stimulus presented to the other eye. The testing stimulus was left/right oriented grating in experiment one, face/house in experiment two, and happy/sad cartoon face in experiment three. Participants made a two-alternative forced choice indicating the identity of the testing stimulus, followed by a perceptual awareness report indicating whether the testing stimulus was seen clearly and discriminable (fully visible), or seen vaguely and un-discriminable (partially visible), or not seen at all (invisible).

The testing stimulus (size: 3 × 3 degree of visual angle) and the masking stimulus (size: 4 × 4 degree of visual angle) were presented in a black background on the two halves of a calibrated CRT monitor (Viewsonic P225, size 22″, spatial resolution of 1024 × 768 pixels, refresh rate of 100 Hz, viewing distance of 81.2 cm). To aid binocular convergence, each stimulus was placed in a white square frame (size: 4 × 4 degree of visual angle) with a red fixation cross at its center, and viewed through a mirror stereoscope with a chin rest. The testing stimulus and the masking stimulus were presented for 300 ms, after which the testing stimulus was replaced by the masking stimulus and the same masking stimulus was presented to the two eyes for 600 ms (Fig. 1). Following the stimulus offset, participants made an unspeeded two-alternative forced choice judgment as to whether the testing stimulus was a left-oriented or a right-oriented grating (experiment one), a face or a house (experiment two), and a happy or a sad face (experiment three). Participants then made an unspeeded perceptual awareness report as to whether the testing stimulus was seen clearly and discriminable (fully visible), or seen vaguely and un-discriminable (partially visible), or not seen at all (invisible).

To control the physical differences between different testing stimuli, we adjusted them to have the same mean luminance and the same luminance distribution. Thus, any difference in discrimination accuracy was not the artifact of some testing stimuli being physically more similar and consequently less discriminable. The mean luminance of the testing stimulus was 4.75%, 9.5%, 19%, 28.5%, 38% of the maximum luminance of the monitor (2.4 cd/m^2^, 4.8 cd/m^2^, 9.7 cd/m^2^, 14.5 cd/m^2^, 19.4 cd/m^2^). The mean luminance of the masking stimulus was 50% of the maximum luminance (25.5 cd/m^2^). For each experiment, participants completed 1250 trials that were divided into 5 blocks of 250 trials (5 luminance values x 50 trials). Within each experiment, the luminance and the identity of the testing stimulus was randomized but counter-balanced across trials. The order of the experiments was counter-balanced across participants.

### Data Analysis

In each experiment, the trials of each luminance scale were divided into three sets where the testing stimulus was invisible, partially visible, and fully visible, respectively. For each of these sets, we calculated the discrimination accuracy (i.e., the percentage of correct answers), and the percent invisible (i.e., the percentage of trials in which the testing stimulus was invisible). The percent invisible reflected the effectiveness of the masking stimulus. It decreased with the luminance of the testing stimulus. In particular, the percent invisible for the five luminance levels was [96% ± 1.5%, 77% ± 2.1%, 54% ± 2.1%, 21% ± 2.7%, 11% ± 0.6%] (mean ± SEM) in experiment one, [98% ± 0.7%, 74% ± 2.3%, 49% ± 3.2%, 26% ± 3.1%, 12% ± 1.1%] (mean ± SEM) in experiment two, and [99% ± 0.5%, 75% ± 3.8%, 48% ± 1.9%, 22% ± 2.9%, 11% ± 0.8%] (mean ± SEM) in experiment three. This proximity in percent invisible across different testing stimuli (i.e., different experiments) allowed us to study the influence of stimulus complexity on the extent of unconscious processing.

## Results

We performed a three-way analysis of variance test (ANOVA) with the stimulus visibility, the stimulus luminance, and the stimulus complexity as three factors-of-interest. We found that the discrimination accuracy of the testing stimulus varied with the stimulus visibility (F(2, 42) = 351.8, p < 10^−13^), the stimulus luminance (F(4, 40) = 75.9, p < 10^−9^), and the stimulus complexity (F(2, 42) = 15.7, p < 10^−3^). Moreover, the analysis revealed a significant interaction between the stimulus visibility and the stimulus luminance (F(8, 36) = 23.13, p < 10^−6^), as well as a significant interaction between the stimulus visibility and the stimulus complexity (F(4, 40) = 4.65, p < 0.05), but no interaction between the stimulus luminance and the stimulus complexity (F(8, 36) = 1.15, p = 0.39). These results suggested that both the luminance and the complexity of the testing stimulus influenced the discrimination accuracy, whereas the exact pattern of influence might differ across different conditions of stimulus visibility.

We therefore explored the exact dependency of the discrimination accuracy on the luminance and the complexity of the testing stimulus, and addressed whether this pattern of dependency varied with the visibility of the testing stimulus. We first investigated how the overall discrimination accuracy changed with the visibility of the testing stimulus. We found that without awareness of the stimulus existence, participants could nevertheless reach above-chance accuracy (>50%) in discriminating the testing stimulus, regardless of its luminance or complexity (t-test; experiment one: t(11) = 44.2, p < 10^−13^; experiment two: t(11) = 35.1, p < 10^−11^; experiment three: t(11) = 57.1, p < 10^−14^). This discrimination accuracy of invisible testing stimulus, however, was significantly lower than the discrimination accuracy where the same testing stimulus was partially visible (t-test; experiment one: t(11) = 10.7, p < 10^−6^; experiment two: t(11) = 6.7, p < 10^−4^; experiment three: t(11) = 8.1, p < 10^−5^) or fully visible (t-test; experiment one: t(11) = 18.6, p < 10^−8^; experiment two: t(11) = 18.1, p < 10^−8^; experiment three: t(11) = 33.1, p < 10^−11^). Moreover, the discrimination accuracy of partially visible stimulus was lower than the discrimination accuracy of fully visible stimulus (t-test; experiment one: t(11) = 3.1, p < 0.01; experiment two: t(11) = 3.3, p < 0.01; experiment three: t(11) = 5.1, p < 10^−3^). These results suggested that participants could form unconscious knowledge of subliminal visual information, although not as accurate as the conscious knowledge of supraliminal visual information.

**Figure 2:**
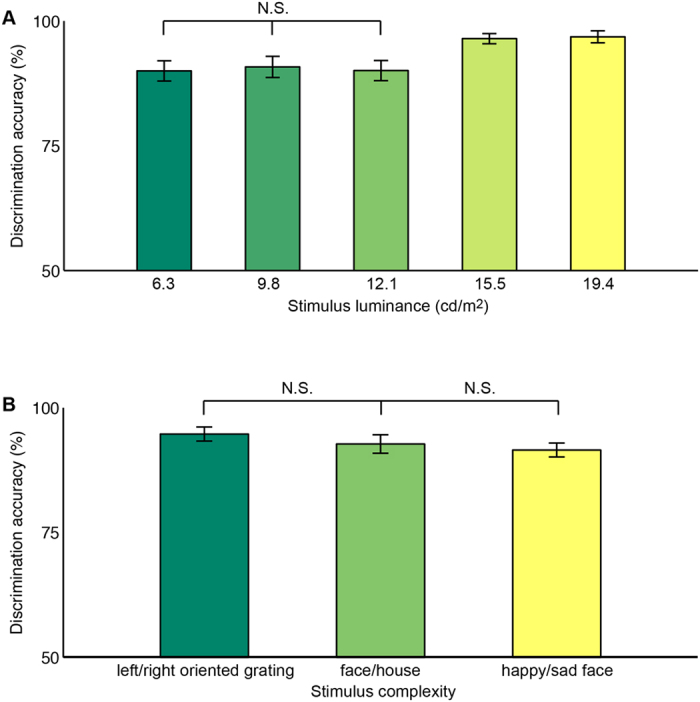
Discrimination accuracy of visible testing stimulus. The discrimination accuracy of testing stimulus was plotted against stimulus luminance (**A**) and stimulus complexity (**B**), for trials where the testing stimulus was visible (including both partially visible and fully visible). ANOVA test and t-test with FDR correction for multiple-comparison were performed. The analysis revealed no significant change in discrimination accuracy with stimulus luminance, and no significant change in discrimination accuracy from left/right oriented grating to face/house or to happy/sad cartoon face. Error bars represent one SEM (N = 12).

We then plotted the discrimination accuracy of the testing stimulus against the stimulus luminance and the stimulus complexity, separately for different conditions of stimulus visibility. In trials where the testing stimulus was partially or fully visible, we did not observe a significant dependency of the discrimination accuracy (averaged across stimuli with different complexity) on the stimulus luminance (Fig. 2A; one-way ANOVA with FDR correction for multiple-comparison; partially visible, F(2, 33) = 4.1, p = 0.08; fully visible, F(2, 33) = 1.1, p = 0.81). Moreover, the discrimination accuracy (averaged across stimuli with different luminance) did not change significantly from left/right oriented grating to face/house (Fig. 2B; t-test with FDR correction for multiple-comparison; partially visible, T(11) = 0.9, p = 0.36; fully visible, T(11) = 0.6, p = 0.81), or from left/right oriented grating to happy/sad cartoon face (Fig. 2B; t-test with FDR correction for multiple-comparison; partially visible, T(11) = 2.1, p = 0.09; fully visible, T(11) = 0.1, p = 0.91).

**Figure 3:**
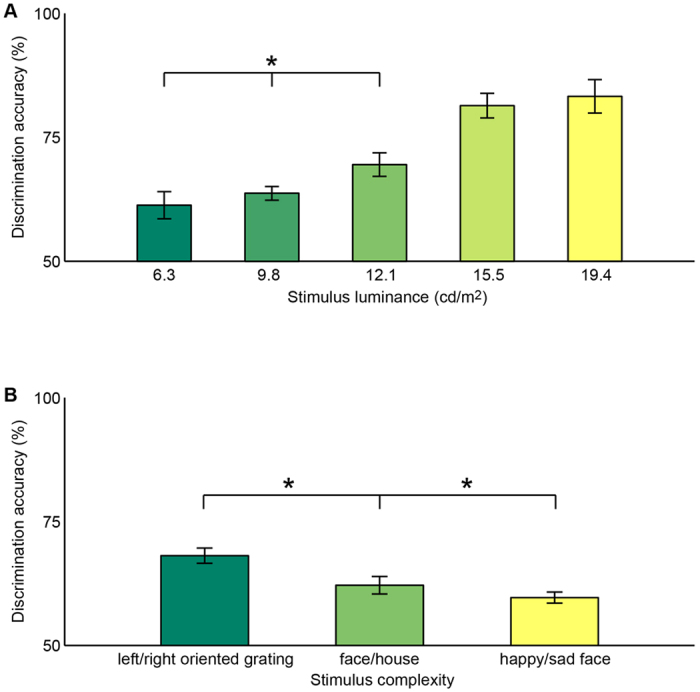
Discrimination accuracy of invisible testing stimulus. The discrimination accuracy of testing stimulus was plotted against stimulus luminance (**A**) and stimulus complexity (**B**), for trials where the testing stimulus was invisible. ANOVA test and t-test with FDR correction for multiple-comparison were performed. The analysis revealed a significant increase in discrimination accuracy with stimulus luminance, and a significant decrease in discrimination accuracy from left/right oriented grating to face/house or to happy/sad cartoon face. Error bars represent one SEM (N = 12).

By contrast, in trials where the testing stimulus was invisible, the discrimination accuracy (averaged across stimuli with different complexity) increased with the stimulus luminance, even when the stimulus luminance changed mildly from level one to level three (Fig. 3A; one-way ANOVA with FDR correction for multiple-comparison; F(2, 33) = 17.8, p < 10^−4^). Moreover, the discrimination accuracy (averaged across stimuli with different luminance) decreased from left/right oriented grating to face/house (Fig. 3B; t-test with FDR correction for multiple-comparison; T(11) = 4.8, p < 10^−3^) or happy/sad cartoon face (Fig. 3B; t-test with FDR correction for multiple-comparison; T(11) = 5.8, p < 10^−3^). These results suggested that the extent of unconscious processing was dependent on the strength and the complexity of the signal carrying subliminal visual information.

## Discussion

Unconscious processing of subliminal visual information was first observed in blindsight patients^7^,^14^,^15^. Later on, by using transcranial magnetic stimulation (TMS) to create artificial scotoma^16^ or using binocular rivalry to render monocular stimulus invisible^8^, it was found that healthy human participants could also reach above-chance accuracy in discriminating invisible visual stimuli. Importantly, such subliminal visual information influences behavior in a fashion similar to the influenced exerted by supraliminal visual information. For example, subliminal visual stimulus can induce visual illusion just as supraliminal visual stimulus does^1^,^2^,^17^. Moreover, the influence of subliminal visual stimulus is not limited to low-level sensory domains but also evident in high-level cognitive domains, where subliminal stimulation of achievement-related words was found to influence goal pursuits and improve task performances^3^,^5^,^6^,^18^,^19^.

The widespread influence of subliminal visual information raises the question of what is its limit. One way to address this question is to test the extent towards which participants can unconsciously process subliminal visual stimulus. The extent of such unconscious processing is likely to be affected by the strength and the complexity of the signal carrying subliminal visual stimulus and may in turn indicate the degree towards which subliminal visual stimulus can influence behavior. As such, studying how the extent of unconscious processing depends on stimulus properties is an important step towards understanding unconscious processing of subliminal visual stimulus. Whereas the existence of unconscious processing of subliminal visual stimulus is well established, its dependence on stimulus properties remains unclear.

Here we explored how the extent of unconscious processing depends on the properties of subliminal visual stimulus. It is plausible that subliminal visual stimulus of different complexity or from different categories was processed by visual cortical regions at different hierarchical levels^20^,^21^,^22^. As such, we compared simple, low-level visual stimulus (e.g., oriented grating) with complex, high-level visual stimulus (e.g., face or house), in order to explore how the complexity of the signal carrying subliminal visual stimulus might affect the extent of unconscious processing. Moreover, we used visual stimulus with different luminance^23^, in order to explore how the strength of the signal carrying subliminal visual stimulus might affect the extent of unconscious processing. We found that the extent towards which participants processed subliminal visual stimulus (i.e., the discrimination accuracy of invisible testing stimulus) increased with stimulus luminance and decreased with stimulus complexity. These results suggested that the strength and the complexity of the signal carrying subliminal visual stimulus affected the extent of unconscious processing. It will be of interest for future studies to address, using neuroimaging techniques, the exact cortical processing for subliminal visual information.

## Additional Information

**How to cite this article**: Song, C. and Yao, H. Unconscious processing of invisible visual stimuli. *Sci. Rep.* **6**, 38917; doi: 10.1038/srep38917 (2016).

**Publisher's note:** Springer Nature remains neutral with regard to jurisdictional claims in published maps and institutional affiliations.
